# The numbers of Libyan doctors in diaspora: Myths and facts

**DOI:** 10.3402/ljm.v7i0.19766

**Published:** 2012-10-19

There is a widely held belief among Libyan medical professionals and the general Libyan public that the number of Libyan doctors in diaspora is around 4,000–5,000. Recently, a Libyan official stated in a television debate that about 5,000 Libyan doctors are scattered around the world. The literature does not support this belief (1–6).

There are only three articles containing data about Libyan doctors in diaspora. Clemens and Pettersson reported a total of 585 Libyan physicians when they used the destination-country census data of 2000 to estimate the number of African-born doctors who had immigrated to the United Kingdom (UK), the United States of America (USA), Australia, Canada, France, Spain, and Belgium (1). Mullan, when examining physician immigration to the UK, the USA, Australia, and Canada in 2004, reported that 624 Libyan doctors were practising in these countries and that 63% of them were in the UK (2). Mullan estimated that 8.9% of all Libyan physicians were practicing in the USA, the UK, Canada, or Australia (2). Moreover, when Arah calculated physician migration density (3), that is, the number of migrating physicians per 1,000 population, Libya was among the top five African countries.

In December 2011, I contacted the General Medical Council (GMC) in the UK, who confirmed that 707 doctors registered in the UK had obtained their primary medical qualification in Libya (4). Of these, 211 doctors are registered in the GMC's Specialist Register (eligible as consultants) and 20 are in the General Practitioner Register (5).

Based on the above-mentioned data, the number of Libyan doctors in the UK, the USA, Australia, and Canada in 2011 can be estimated at 1,120 (707 in the UK (4), which, according to Mullan (2), is home to 63% of the total number of Libyan doctors in these four countries). The number of doctors emigrating from Libya every year can also be estimated from the 707 doctors in the UK in 2011 and 393 doctors in the UK in 2004 [from (2)]. This means 314 doctors have immigrated to the UK over 7 years, which averages to 45 doctors per year. With the UK being home to 63% of Libyan doctors in the UK, the USA, Australia and Canada, the immigration rate to these countries combined is about 71 doctors per year.

These data have several limitations. First, they do not include Libyan doctors who are practising in the Arab Gulf states, such as Saudi Arabia, Qatar, and the United Arab Emirates. However, most, if not all, Libyan doctors in the Gulf States did their postgraduate training in Western countries and probably kept their registration valid with the official bodies in these countries, hence they are likely to be included in the published studies. Second, some official bodies, such as the GMC in the UK, keep data on the country in which the primary medical degree was obtained, not on nationality. Therefore, the minority of Libyan doctors who did not obtain their primary degrees from Libya are not included in these databases. On the other hand, non-Libyans who obtained their primary medical degree from Libya and settled in the West are included. However, the numbers in both latter groups are probably too small to substantially affect estimates of the total number of Libyan doctors in diaspora. Third, some Libyan doctors who are working towards an academic degree, such as Master's or PhD, will also not appear in the official registers. Most of these doctors will probably return home after obtaining their degrees because academic positions are very limited. Fourth, Libyan doctors who are practising in other European countries such as Sweden and Italy are also not included in the available data. However, their number is too small to have a significant impact. Finally, the data from the British GMC might give the impression that most of the Libyan doctors in the UK are not trained to consultant level (69%, 476 out of 687). However, the majority of Libyan doctors in the UK who are not registered consultants are experienced clinicians and many of them have worked for years as locum consultants. It should also be noted that of the 69%, some doctors are still in clinical training.

Therefore, according to the literature, the belief that the number of Libyan doctors in diaspora is about 4,000–5,000 is a *myth*. There are probably no more than 1,250–1,500 Libyan doctors practising outside Libya. Nevertheless, the return of many of these doctors to Libya will help in building an efficient health care system.

*Hani T.S. Benamer* Neurology Department New Cross Hospital Wolverhampton WV10 OQP, United Kingdom

